# Novel *Moraxella catarrhalis* prophages display hyperconserved non-structural genes despite their genomic diversity

**DOI:** 10.1186/s12864-015-2104-1

**Published:** 2015-10-24

**Authors:** Amir Ariff, Michael J. Wise, Charlene M. Kahler, Chin Yen Tay, Fanny Peters, Timothy T. Perkins, Barbara J. Chang

**Affiliations:** School of Pathology and Laboratory Medicine, The University of Western Australia, Perth, WA Australia; School of Chemistry and Biochemistry, The University of Western Australia, Perth, WA Australia

**Keywords:** *Moraxella catarrhalis*, Prophages, Bacteriophages, *Siphoviridae*, Pan-genome, Multi-locus sequence typing, Phage-related genes, Hyperconservation, Gram-negative diplococcus, Non-structural genes

## Abstract

**Background:**

*Moraxella catarrhalis* is an important pathogen that often causes otitis media in children, a disease that is not currently vaccine preventable. Asymptomatic colonisation of the human upper respiratory tract is common and lack of clearance by the immune system is likely due to the emergence of seroresistant genetic lineages. No active bacteriophages or prophages have been described in this species. This study was undertaken to identify and categorise prophages in *M. catarrhalis,* their genetic diversity and the relationship of such diversity with the host-species phylogeny.

**Results:**

This study presents a comparative analysis of 32 putative prophages identified in 95 phylogenetically variable, newly sequenced *M. catarrhalis* genomes. The prophages were genotypically classified into four diverse clades. The genetic synteny of each clade is similar to the group 1 phage family *Siphoviridae*, however, they form genotypic clusters that are distinct from other members of this family. No core genetic sequences exist across the 32 prophages despite clades 2, 3, and 4 sharing the most sequence identity. The analysis of non-structural prophage genes (coding the integrase, and terminase), and portal gene showed that the respective genes were identical for clades 2, 3, and 4, but unique for clade 1. Empirical analysis calculated that these genes are unexpectedly hyperconserved, under purifying selection, suggesting a tightly regulated functional role*.* As such, it is improbable that the prophages are decaying remnants but stable components of a fluctuating, flexible and unpredictable system ultimately maintained by functional constraints on non-structural and packaging genes. Additionally, the plate encoding genes were well conserved across all four prophage clades, and the tail fibre genes, commonly responsible for receptor recognition, were clustered into three major groups distributed across the prophage clades. A pan-genome of 283,622 bp was identified, and the prophages were mapped onto the diverse *M. catarrhalis* multi-locus sequence type (MLST) backbone.

**Conclusion:**

This study has provided the first evidence of putatively mobile prophages in *M. catarrhalis*, identifying a diverse and fluctuating system dependent on the hyperconservation of a few key, non-structural genes. Some prophages harbour virulence-related genes, and potentially influence the physiology and virulence of *M. catarrhalis*. Importantly our data will provide supporting information on the identification of novel prophages in other species by adding greater weight to the identification of non-structural genes.

**Electronic supplementary material:**

The online version of this article (doi:10.1186/s12864-015-2104-1) contains supplementary material, which is available to authorized users.

## Background

*M. catarrhalis* is a commensal and important pathogen of the human upper respiratory tract and middle ear. After *Streptococcus pyogenes* and *Haemophilus influenzae*, *M. catarrhalis* is the leading cause of upper respiratory tract and middle ear infections in humans, commonly presenting as otitis media in children below 2 years of age [1], and as a variety of diseases in adults and the elderly, ranging from chronic obstructive pulmonary disease (COPD), to pneumonia, bronchitis, laryngitis, and sinusitis [2]. Infrequently, *M. catarrhalis* can cause septic arthritis, bacteraemia [3], endocarditis [4], meningitis [5], and other invasive infections [2]. *M. catarrhalis* colonises the upper respiratory tracts of up to 75 % of children, though the colonisation rate reduces to approximately 1 % in adults [2]. *M. catarrhalis* has a range of virulence factors, which allow the bacterium to adhere to host epithelial cells, enter host tissues, successfully multiply, interfere with and avoid host defence mechanisms, and cause disease [2]. The virulence of *M. catarrhalis* is well described, particularly regarding the mechanisms of serum-resistance [6–8]. The characterisation of virulence factors has been associated with two major clades of the bacterium: 1) a serum-resistant clade that has a higher representation of virulence factors, is complement-resistant, has increased adhesion to epithelial cells, displays higher genomic diversity, and has a more recent evolution of about 5 Ma ago; and 2) a serum-sensitive clade, which has the converse traits [9].

Bacteriophages are viruses that infect bacteria and utilise the host’s cell machinery to replicate and propagate. Two lifecycles of phages are defined: lytic phages, which contain a copy of the phage genome packaged in its capsid that is built into its quaternary structure prior to lysing the host cell and subsequent release; and lysogenic or temperate phages, which may opt instead to integrate into the host cell’s genome, lying dormant until conditions are suitable to re-enter the lytic pathway [10]. Temperate phages that are integrated into the host genome are known as prophages. Prophages may harbour cargo genes, which are non-essential for phage function, but may confer virulence and other traits to the host cell. This may benefit either or both the phage and host bacterium, and is well categorised in the case of toxins, cell adhesion molecules, nutrient uptake, immune response evasion, fimbriae and others [11, 12]. While integrated into the host genome, prophages undergo evolutionary pressures different from those undergone by phage particles, leading to host-prophage driven selection and genetic flux, even within prophage genes that do not affect host physiology [13, 14]. The rapid increase in bacterial genome sequencing has led to the identification of numerous prophages [15] and has furthered our understanding of phage roles in host physiology and pathology of disease [16].

Active phages are often difficult to identify due to their size and sporadic signals that lead to lytic activation. Scant phage-associated open reading frames (ORFs) have been described in *M. catarrhalis* genomes [15], however, no active phages or complete prophages have yet been identified in the species. As phages may contribute in important ways to the survival and pathogenesis of *M. catarrhalis*, we aimed to screen a diverse collection of *M. catarrhalis* isolates for the presence of prophages and to characterise these genetic elements. This study utilised a bioinformatics approach, resulting in the identification of 32 novel *M. catarrhalis* prophages. Analysis of the prophage pan-genome and phage-related genes supported the categorisation of prophages into four clades, where clades 2, 3, and 4 share regions of homogeneity and clade 1 was unique. Further analysis of the phage-related genes showed a relationship with known double-stranded DNA, tailed phages, the *Siphoviridae*. The non-structural genes encoding the integrase and terminase, as well as a structural gene encoding the portal protein were hyperconserved despite the surrounding diversity of the identified prophages. Additionally, several virulence-related factors were identified, which may suggest a role of these prophages in virulence of *M. catarrhalis*.

## Results

### Identification of putative *M. catarrhalis* prophages

PHAST identified 32 putative complete prophages, 54 questionable prophages, and 131 incomplete prophages in 95 *M. catarrhalis* genomes (Table 1 and Additional file 1: Figure S1.; see methods for classification criteria). Each of the 32 putative complete prophages was found in a different strain of *M. catarrhalis,* and the strains O35E and 2041717D harboured no prophage of any category.

**Table 1:** Strains analysed in this study

Strain	Source^a^	Clinical/Commensal	MLST^b^	Phage presence^c^			Phage name^d^ (%GC)	*att* site^e^	NCBI prophage accession number
				I	Q	C			
2019228G	PathWest	Clinical	^h^	1	0	0			
1020848 M	PathWest	Clinical	11	2	0	1	Mcat7 (43.62 %)	5’-ATCAAAAAATGG-3’ 5’-AATCAAAAATCT-3’ 5’-TTTTTTATTGGG-3’	KR093631
1028680 K	PathWest	Clinical	41	1	0	0			
1034084Q	PathWest	Clinical	224	1	0	0			
2023641 W	PathWest	Clinical	4	2	1	0			
3331584D	PathWest	Clinical	^h^	0	1	0			
3476642E	PathWest	Clinical	46	1	1	0			
3481088Y	PathWest	Clinical	^h^	2	3	0			
3753746B	PathWest	Clinical	^h^	0	0	1	Mcat9 (43.94 %)	5’-TGTGTACATAATTGTGTACATA-3’	KR093633
4789849 F	PathWest	Clinical	67	1	1	0			
5550565E	PathWest	Clinical	^h^	3	1	1	Mcat32 (42.68 %)	N/A	KR093656
20236154	PathWest	Clinical	236	1	0	0			
20370737	PathWest	Clinical	105	1	0	1	Mcat4 (43.21 %)	5’-TTATCAATCAGT-3’	KR093628
2040048B	PathWest	Clinical	230	0	1	0			
3503282R	PathWest	Clinical	184	2	0	1	Mcat16 (42.96 %)	5’-TTTTTTTAGGGG-3’	KR093640
5004663G	PathWest	Clinical	224	1	0	0			
1093063Y	PathWest	Clinical	230	0	1	0			
4640032P	PathWest	Clinical	246	1	0	1	Mcat15 (42.71 %)	5’-ATACAAAAAATC-3’	KR093639
2046210Y	PathWest	Clinical	^h^	2	0	0			
4737718Q	PathWest	Clinical	^h^	2	0	0			
5012204D	PathWest	Clinical	250	4	2	1	Mcat24 (42.74 %)	N/A	KR093648
4840991 N	PathWest	Clinical	73	1	0	0			
2042044P	PathWest	Clinical	^h^	0	0	0			
2041717D	PathWest	Clinical	^h^	0	0	0			
5008863 L	PathWest	Clinical	^h^	1	1	1	Mcat19 (42.39 %)	5’-ATTTTTTATATT-3’	KR093643
1583718S	PathWest	Clinical	234	2	1	0			
5267783B	PathWest	Clinical	234	3	1	0			
4431503 J	PathWest	Clinical	118	3	2	0			
1111988H	PathWest	Clinical	^h^	1	2	0			
5021467Y	PathWest	Clinical	224	1	0	0			
5560626Q	PathWest	Clinical	^h^	1	0	0			
2047127 K	PathWest	Clinical	240	1	0	0			
5021466 N	PathWest	Clinical	224	1	0	0			
2041417P	PathWest	Clinical	^h^	3	1	1	Mcat3 (43.79 %)	5'-AAAAAAATCAAAG-3’	KR093627
1098655R	PathWest	Clinical	230	1	1	0			
5157102Y	PathWest	Clinical	251	1	0	0			
4849094R	PathWest	Clinical	248	1	0	0			
2050675Y	PathWest	Clinical	^h^	1	0	0			
5553245S	PathWest	Clinical	^h^	1	0	0	Mcat13 (42.77 %)	5’-TTTTTCAGCTTC-3’	KR093637
5/131/1	KOMRP	Commensal	217	1	0	0			
23/41/1	KOMRP	Commensal	235	2	0	0			
39/355/1	KOMRP	Commensal	62	1	1	1	Mcat2 (43.64 %)	5’-TTTCAAATTTTA-3’	KR093626
41/539/1	KOMRP	Commensal	^h^	1	0	1	Mcat22 (42.48 %)	5’-AAAAATTTGGTT-3’	KR093646
3/7/1	KOMRP	Commensal	242	1	1	0			
102/402/3	KOMRP	Commensal	^h^	1	3	0			
91/291/2	KOMRP	Commensal	^h^	2	0	1	Mcat20 (42.71 %)	N/A	KR093644
78/191/1	KOMRP	Commensal	^h^	4	0	1	Mcat31 (42.68 %)	N/A	KR093655
96/281/2	KOMRP	Commensal	259	1	0	0			
26/133/1	KOMRP	Commensal	70	2	0	1	Mcat23 (42.65 %)	N/A	KR093647
129/414/4	KOMRP	Commensal	209	1	0	0			
130/563/4	KOMRP	Commensal	74	2	0	0			
60/120/1	KOMRP	Commensal	64	1	0	1	Mcat11 (43.92 %)	5’-TAAAAAAAATAAA-3’	KR093635
105/305/2	KOMRP	Commensal	^h^	0	1	0			
113/391/3	KOMRP	Commensal	232	1	0	0			
20/122/1	KOMRP	Commensal	235	2	0	0			
79/220/2	KOMRP	Commensal	^h^	1	2	0			
29/50/1	KOMRP	Commensal	241	0	1	0			
23/95/1	KOMRP	Commensal	233	1	0	0			
107/374/3	KOMRP	Commensal	229	2	0	0			
76/204/3	KOMRP	Commensal	^h^	1	0	0			
73/187/1	KOMRP	Commensal	254	0	1	1	Mcat10 (44.14 %)	5’-TAAAAAAAATAAA-3’	KR093634
24/92/1	KOMRP	Commensal	64	0	0	1	Mcat12 (43.56 %)	5’-TTATTTTTAAAA-3’	KR093636
78/325/3	KOMRP	Commensal	256	2	0	0			
78/205/2	KOMRP	Commensal	^h^	5	0	1	Mcat30 (42.68 %)	N/A	KR093654
105/417/3	KOMRP	Commensal	^h^	1	1	0			
25/44/1	KOMRP	Commensal	^h^	1	1	0			
77/338/2	KOMRP	Commensal	50	0	2	0			
16/38/1	KOMRP	Commensal	157	1	0	1	Mcat26 (42.22 %)	N/A	KR093650
64/108/1	KOMRP	Commensal	140	0	2	1	Mcat17 (42.46 %)	5’-ACCATTTTTTAA-3’ 5’-TTTTTTTCATTTT-3’	KR093641
3/22/1	KOMRP	Commensal	242	1	0	0			
80/196/2	KOMRP	Commensal	^h^	2	1	0			
15/36/1	KOMRP	Commensal	233	1	0	0			
BBH18	Sputum sample, patient with COPD, Holland GCA_000092265.1	Clinical	128	1	0	0			
A16	Frozen stocks, saliva, Australia	Commensal	^h^	0	0	0	Mcat28 (42.24 %)	5’-TGTGTACATAATTGTGTACATA-3’	KR093652
								5’-AAAAAACTTAAC-3’	
ATCC43617^f^	Trans-tracheal aspirate from coal miner with bronchitis	Clinical	25	1	1	1	Mcat5 (43.30 %)	N/A	KR093629
ATCC43617a^f^	Trans-tracheal aspirate from coal miner with bronchitis	Clinical	25	1	1	1	Mcat6 (43.08 %)	N/A	KR093630
BE4L	PathWest	Clinical	^h^	2	0	1	Mcat25 (42.61 %)		
MC4S	Alexander project	Clinical	188	1	2	1	Mcat8 (41.40 %)	5’-CTTAAAAAAATA-3’	KR093632
MC24	Alexander project	Clinical	^h^	1	0	0			
MC1	Alexander project	Clinical	131	1	0	0			
BE5	PathWest	Clinical	^h^	1	1	1	Mcat18 (42.26 %)	5’-CATTAATCAAAT-3’	KR093642
A6	Frozen stocks, saliva, Australia	Commensal	3	6	0	1	Mcat29 (42.23 %)	N/A	KR093653
T6	Sputum, children 3 – 6 years, Taiwan	Commensal	^h^	1	1	0			
T12	Sputum, children 3 – 6 years, Taiwan	Commensal	^h^	2	1	0			
T4	Sputum, children 3 – 6 years, Taiwan	Commensal	191	1	0	0			
K117	Hospital isolate, Perth, Australia	Clinical	40	1	0	0			
12P80B1^g^	GCA_000192965.2	Clinical	185	1	0	0			
O35E^g^	GCA_000193085.2	Clinical	146	0	0	0			
46P47B1^g^	GCA_000192945.2	Clinical	^h^	1	0	1	Mcat21 (42.26 %)	N/A	KR093645
101P10B1^g^	GCA_000193065.2	Clinical	218	1	1	0			
103P14B1^g^	GCA_000192925.2	Clinical	187	1	1	1	Mcat14 (43.39 %)	N/A	KR093638
7169^g^	GCA_000192905.2	Clinical	82	3	2	0			
BC1^g^	GCA_0000192985.2	Clinical	216	2	1	0			
BC7^g^	GCA_000193005.2	Clinical	217	1	0	0			
BC8^g^	GCA_0000193025.2	Clinical	162	1	1	1	Mcat1 (43.62 %)	5’-TTTCAAATTTTA-3’	KR093625
CO72^g^	GCA_000193045.2	Clinical	199	2	0	1	Mcat27 (42.57 %)	5’-TGTGTACATAATTGTGTACATA-3’	KR093651
RH4^g^	GCA_000302495.1	Clinical	^h^	0	0	1			

^a^Source of collected strain (PathWest: strains obtained from PathWest Laboratory Medicine WA, Australia; KOMRP: strains obtained from the Kalgoorlie Otitis Media Research Project [49])

^b^Multi locus sequence typing strain types

^c^Phage presence as annotated by PHAST, where I = incomplete prophage, Q = questionable prophage, and C = complete prophage

^d^Complete prophages were named Mcat1 to Mcat32

^*e*^ *att* sites are putative flanking attachment sites identified in *M. catarrhalis* genomes by PHAST. Prophages without identified *att* sites are labelled N/A

^f^The strain ATCC43617a is a passaged derivative of ATCC43617 that was obtained from The University of Queensland and sequenced in this study. Additionally, the sequenced genome of ATCC43617 available on the NCBI database was also analysed [15]

^g^Strains not sequenced in this study, but are available on the NCBI database; GenBank accession numbers current at time of manuscript submission [15]

^h^denotes novel strain types

The putative complete prophages ranged between 25 kb to 55 kb in length, with a median length of approximately 40 kb. The GC content of the prophages ranged between 41.40 and 44.14 %, with an average GC content of 42.90 % - higher than the average GC content for the *M. catarrhalis* host of ~41 % [17]. Twenty-three putative attachment sites were identified amongst 20 prophages (Table 1).

### Classification and similarity of *M. catarrhalis* prophages

**Fig. 1:**
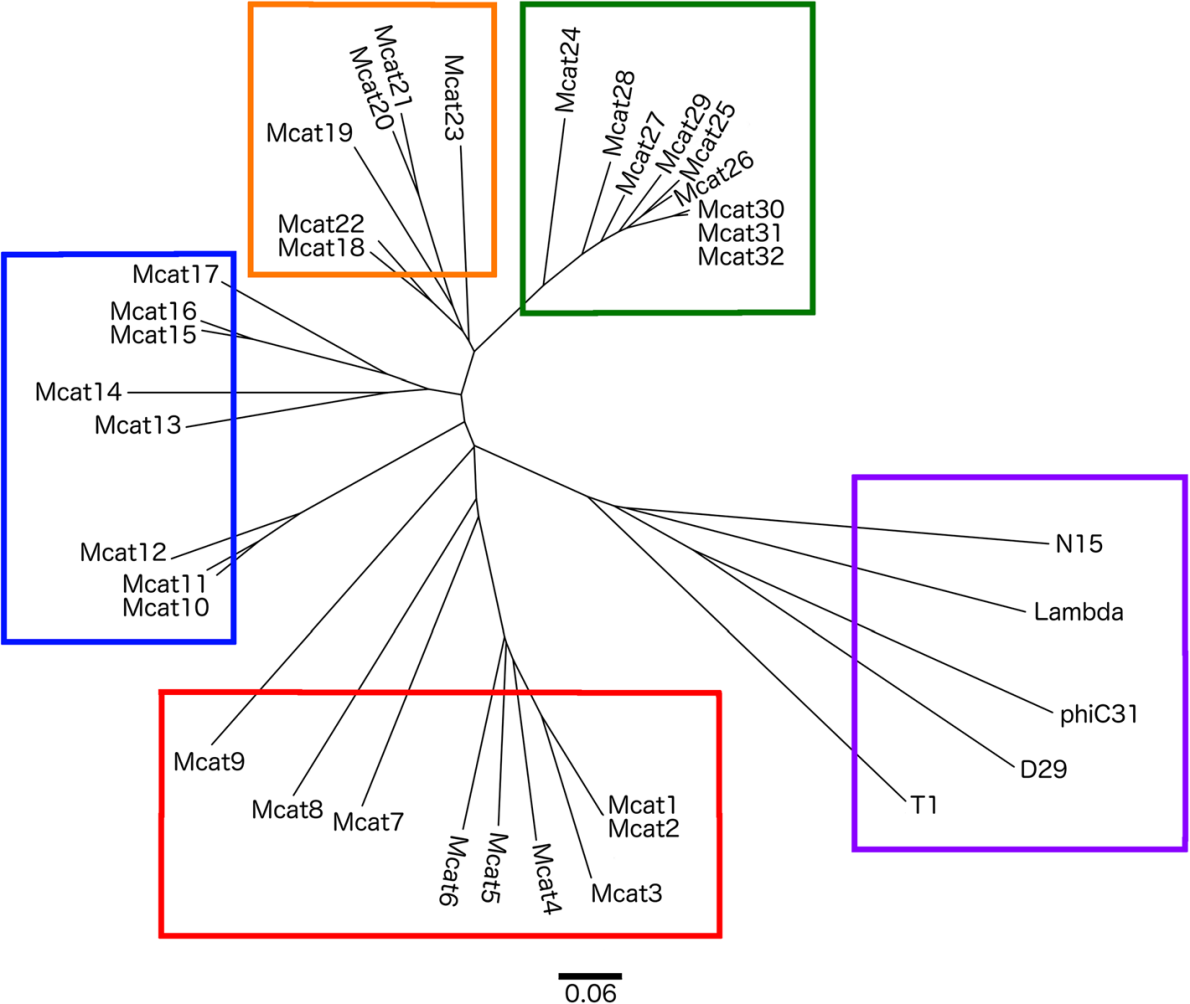
Prophages of *Moraxella catarrhalis* are divided into four clades, grouped by nucleotide similarity. The 32 prophages identified in 95 sequences of *M. catarrhalis* were aligned using progressiveMauve and compared to five reference *Siphoviridae* genera: λ-like, L5-like, N15-like, ϕC31-like, and Tuna-like viruses (purple) and their similarities represented by the Mauve output guide tree. The *M. catarrhalis* prophages are grouped into four clades (red = clade 1, blue = clade 2, orange = clade 3, green = clade 4)

The progressiveMauve blocks alignment categorised the complete putative prophages into 4 distinct clades (Fig. 1). The addition of archetypes from all genera of Caudovirales bacteriophages to the distance tree revealed that *M. catarrhalis* prophages are related to, but distinct from the following genera of *Siphoviridae*: λ-like viruses, L5-like viruses, N15-like viruses, ϕC31-like viruses, and Tuna-like viruses. The archetypes from each of these five genera formed a clade separate from the *M. catarrhalis* prophages. There was no significant identity between the *M. catarrhalis* prophages and the phages of the families *Podoviridae* or *Myoviridae* (data not shown). Additionally, the regions with greatest sequence identity to the listed five genera of *Siphoviridae,* comprised the tail fibre genes. This result was further confirmed by the Virfam analysis, which categorised all prophages as Virfam Type 1 *Siphoviridae* from Virfam Clusters 1, 3, 4 or 5 (Additional file 2: Table S1) [18]. All prophages from clades 2, 3, and 4 were categorised as Type 1, Cluster 3 *Siphoviridae*, with similarity to phages D3, HK97 and HK022, with the exception of Mcat28, which was categorised as a Type 1, Cluster 1 *Siphoviridae* most similar to the SPP1 group of phages. The prophages of clade 1 were more variably categorised, with Mcat1, Mcat2, Mcat3, Mcat4, Mcat7, and Mcat9 being categorised as Type 1, Cluster 3 *Siphoviridae* most similar to prophage ϕ4795; Mcat 5 was categorised as a Type 1, Cluster 4 *Siphoviridae* most similar to phage PBl1; Mcat6 was categorised as a Type 1, Cluster 1 *Siphoviridae* most similar to the SPP1 group of phages; and Mcat8 was categorised as a Type1, Cluster 5 *Siphoviridae* similar to phages such as ϕC31, ϕBT1, and ϕHSIC.

**Fig. 2:**
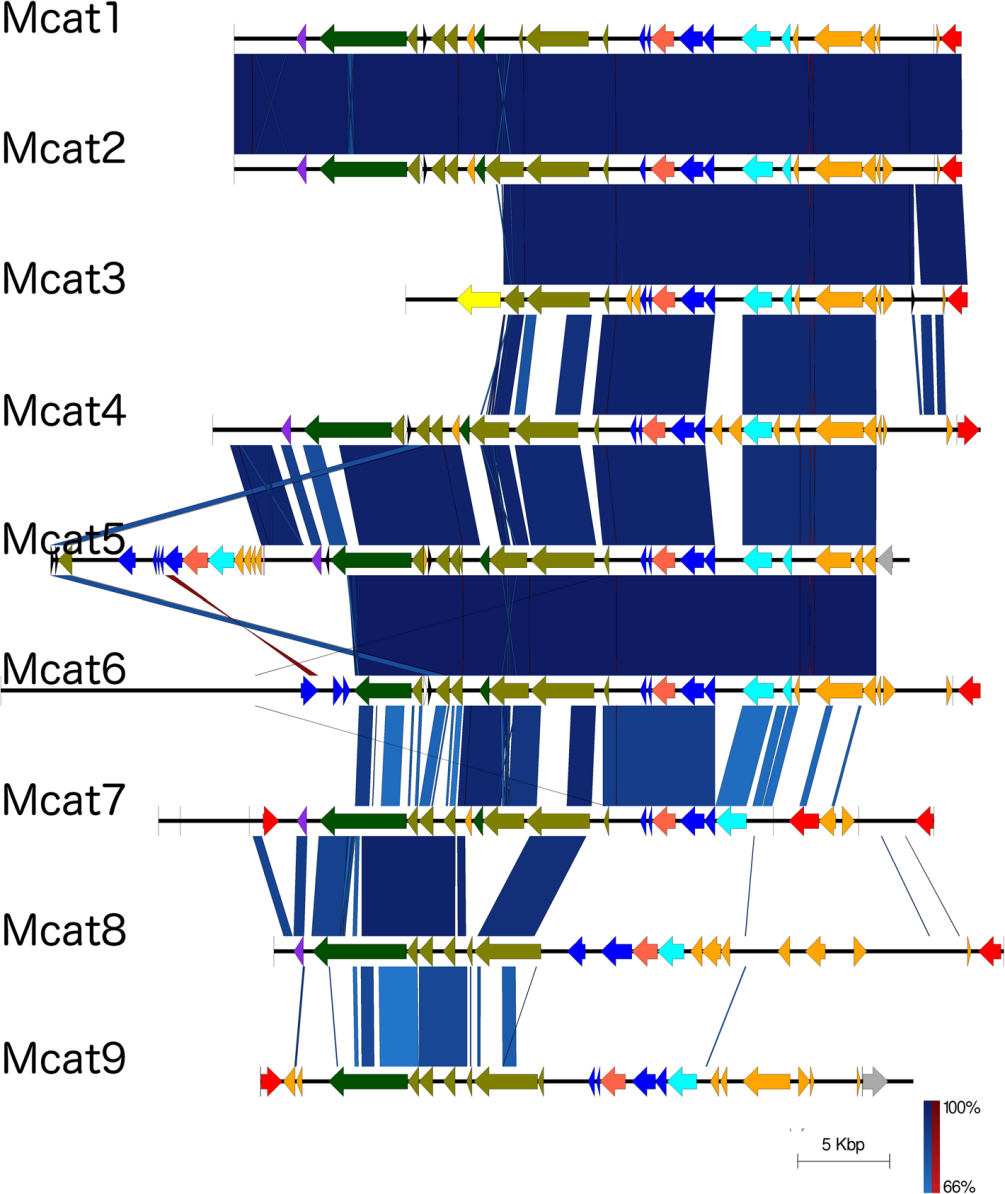
Alignment of *M. catarrhalis* prophages in clade 1. Phage-related genes are shown as arrows to indicate their direction relative to the prophage sequence, and are colour coded as follows: integrase (red), terminase (light blue), coat (dark blue), portal (dark orange), tail shaft (light green), tail fibre (dark green), plate (purple), and protease (yellow). Vertical blue lines between prophages show conserved areas without inversion, and red lines between prophages show conserved areas with inversion; the intensities of these lines correspond to percent identity between adjacent prophages as indicated by the scale (lower right)

Alignment of the *M. catarrhalis* prophages of each clade showed that clades 2, 3, and 4 were well conserved with regards to the sequence identity of phage-related genes, overall phage genomes, and synteny of genes (Additional file 3: Figure S2). Clade 1 was shown to be more variable, especially the prophages Mcat7, Mcat8, and Mcat9 (Fig. 2).

The synteny of putative *M. catarrhalis* prophages is very similar to that of the five reference genera: from proximal to distal, the phage genes are encoded in the same direction, comprising coat protein genes with associated packaging proteins, particularly terminase and portal protein genes, followed by the structural genes encoding tail sheath, tail fibre, and plate or endolysin. Additionally, the integrase gene is located at either end of the prophage. The *M. catarrhalis* clade 1 prophages are characterised by a proximal integrase, followed by the terminase, coat, then portal genes. Clade 2 prophages are different in that the integrase is located distally and is in the reverse coding direction compared to other genes. Clade 3 and 4 prophages have a distal, inverted integrase, subsequently encoding the terminase genes, portal gene, followed by coat genes.

### Analysis of prophage genes

The following categories of genes were identified using PHAST, and used for analysis: coat proteins, integrases, plate proteins, portal proteins, tail (sheath) proteins, tail fibre proteins, and terminase proteins (Additional file 4: Table S2). Additionally, proteases were also identified, but were not used for prophage comparison because only two were identified.

### Highly conserved phage-related genes: integrase, plate, portal, terminase and tail fibre genes

**Fig. 3:**
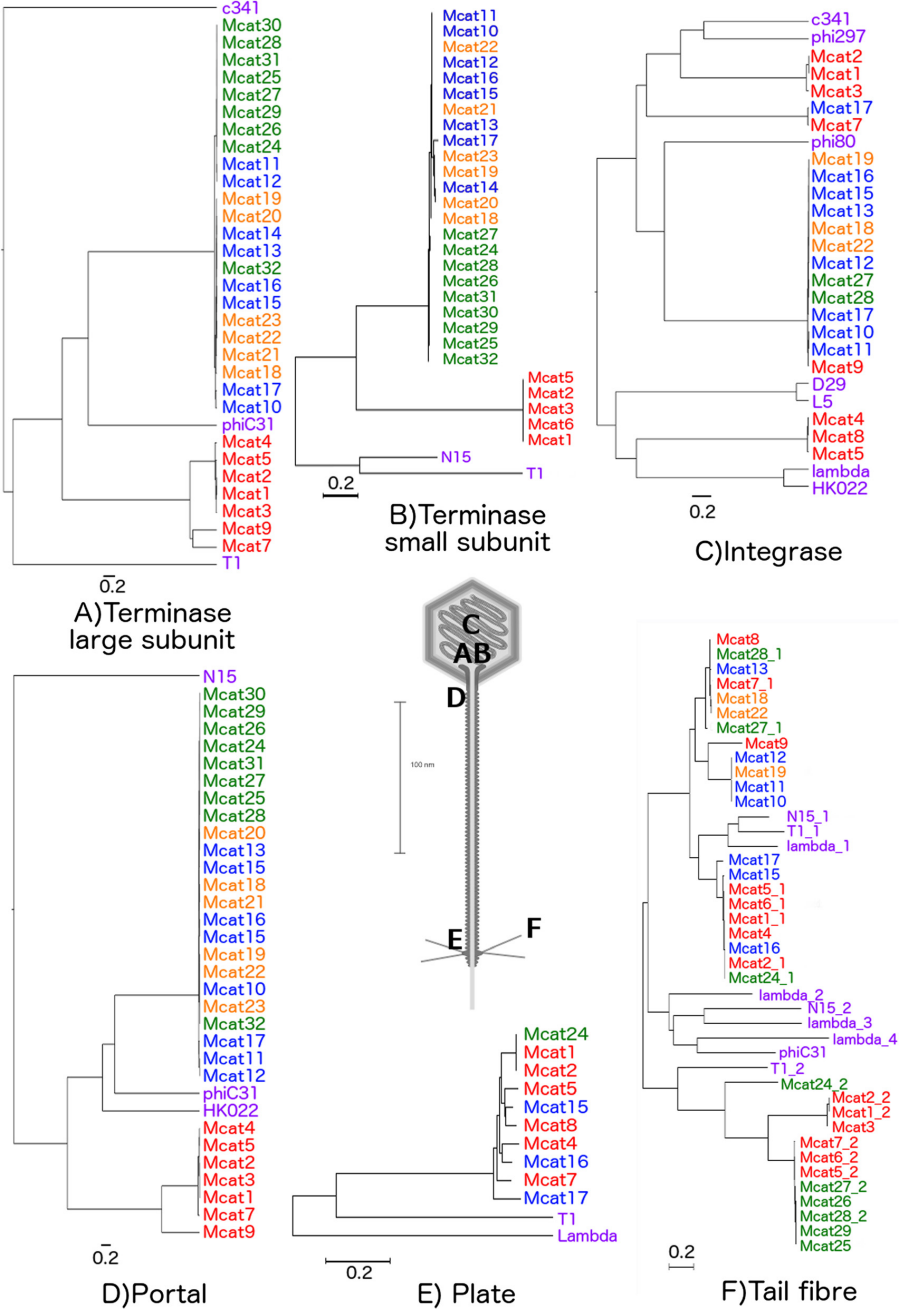
Phage-related gene analysis of *M. catarrhalis* prophages. Distance trees displaying the diversity of phage-related genes in *M. catarrhalis* prophages. **a** Large terminase subunit. **b** Small terminase subunit. **c** Integrase. **d** Portal. **e** Plate. **f** Tail Fibre. The *M. catarrhalis* prophage names are coloured according to clade assignment in Fig. 1; red = clade 1, blue = clade 2, orange = clade 3, green = clade 4. Reference phage genes, as detailed in the methods section, are coloured purple. Scale at lower left of each panel corresponds to 0.2 nucleotide substitutions per site. The image of a *Siphoviridae* phage presented in the centre with 100 nm scale [66] is labelled according to the analysed genes. The **a** terminase large subunit, **b** terminase small subunit, and **c** integrase proteins are found inside the capsid, whereas the **d** portal, **e** plate, and **f** tail fibre are structural

The integrase, plate, portal and terminase genes of *M. catarrhalis* prophages are highly conserved, and a diversity of less than 0.1 substitutions per nucleotide position was used to differentiate between gene clades. In the case of tail fibre genes, a cut-off value of about 0.4 substitutions per nucleotide position yielded better resolution and discrimination power for the clade classification (Fig. 3).

**Fig. 4:**
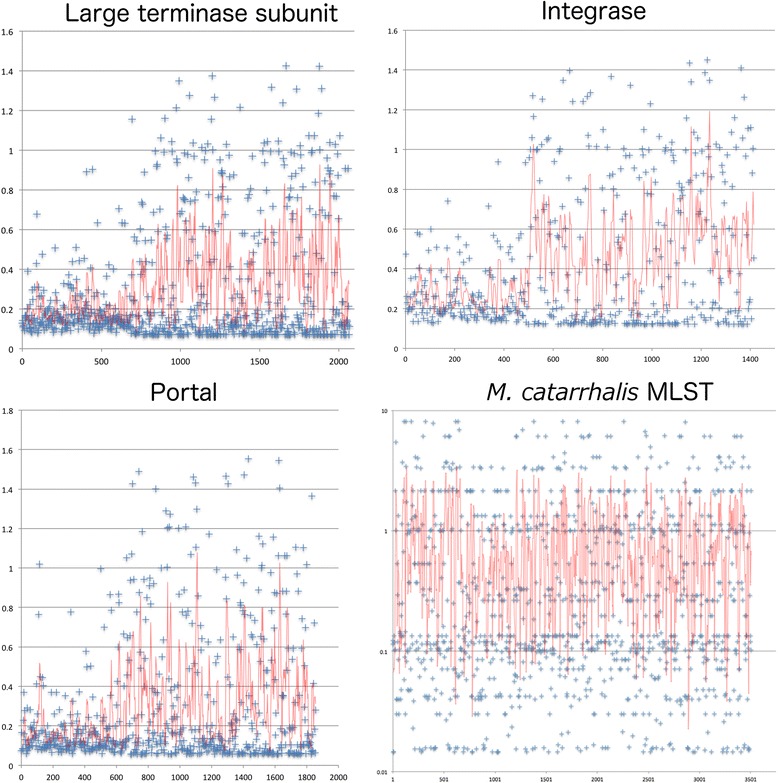
Codon-based measure of evolutionary pressure on *M. catarrhalis* prophage genes. The d_N_/d_S_ value for each codon (represented by each blue +) is plotted against the length of each prophage gene encoding large terminase subunit, integrase, and portal. Similarly, the d_N_/d_S_ value for each codon is plotted for the concatenation of 7 *M.* catarrhalis MLST housekeeping genes. An average value for four subsequent codons is represented by the red line. Values of d_N_/d_S_ < 1 indicates purifying selection, d_N_/d_S_ > 1 indicates diversifying selection, and d_N_/d_S_ = 1 indicates neutrality

These similarity trees show that the phage-related genes of integrase, portal, large terminase subunit and small terminase subunit were distributed according to a trend that indicates a single class of such genes for the clade 1 prophages, which is separate from the respective genes from prophages in clades 2, 3, and 4. This trend was not observed for the plate genes, which seemed to be uniformly distributed for all *M. catarrhalis* prophage classes, and the tail fibre genes, which did not seem to follow any pattern based on prophage clade (Fig. 3). All diversities represented in these trees were confirmed by inferring selective pressure using non-synonymous to synonymous ratios (d_N_/d_S_), which confirm hyperconservation of the integrase, terminase, and portal genes (Fig. 4). Each gene displayed an average d_N_/d_S_ of 0.431, 0.313, and 0.301 respectively, which corresponds to Yates’s corrected *χ*^2^ values of 45.2, 83.8, and 76.8, all of which are statistically significant to the 5 % level for a one-tailed test using the null hypothesis that d_N_/d_S_ values are representative of neutral evolution (H_0_: d_N_/d_S_ = 1). These values are also statistically significant to the 5 % level when compared to the average d_N_/d_S_ value of *M. catarrhalis* MLST housekeeping genes, 0.84 (Fig. 4).

### Phage-related genes of high diversity: coat and tail shaft proteins

The coat and tail shaft genes followed a very complex distribution, because there is more than one gene of each category for each analysed phage. An analysis of the translated nucleotides was used instead, allowing for manual alignment of codons.

In the case of coat genes, comparing the generated clusters with genes of known function in the reference *Siphoviridae* phages revealed seven clusters of genes, four of whose functions could be inferred. These four clusters were grouped according to the following functions: coat adaptor proteins, joining and completion proteases, coat connector proteins, and head proteases. Of the remaining three clusters, only one cluster had a significant size, but with no known reference genes. Another cluster contained two λ coat genes involved in packaging and head-tail joining. The last cluster consisted of a Mcat7 protein and a Mcat9 coat protease (Additional file 5: Figure S3).

Tail shaft genes were more diverse than coat genes, and using a translated nucleotide alignment, some genes from Mcat29, Mcat28 and Mcat25 were so divergent that they returned negative bootstrap values when aligned by ClustalW. Including these data points, up to 13 clusters were found (though some were single data points), of which only five were significant. Four clusters contained reference genes from λ, T1, N15, ϕC31, and D29; of which one cluster contained genes from only these reference phages, without data points from any *M. catarrhalis* prophages. One of the clusters contained only *M. catarrhalis* prophage genes without any reference genes included. Unlike the coat genes, no significant pattern was observed in the tail shaft gene distribution.

### Putative virulence-related genes

**Fig. 5:**
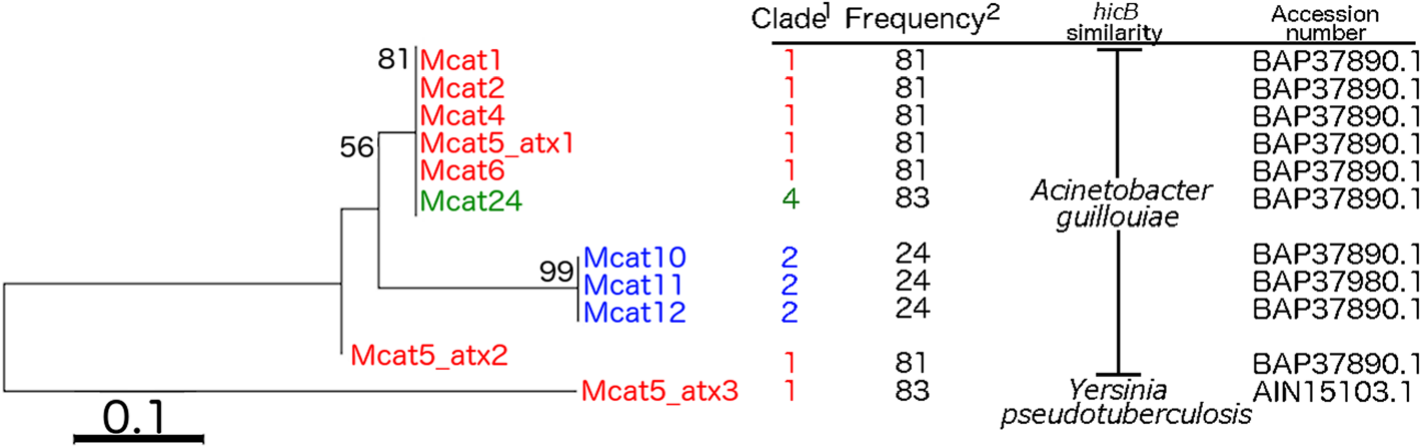
Similarity of antitoxins found in *M. catarrhalis* prophages. Eleven antitoxin components were identified in 9 *M. catarrhalis* prophages with their putative evolutionary relationship depicted above. ^1^The antitoxins are found in prophages contained in clades 1 (red), 2 (green), and 4 (blue). ^2^The frequency at which the respective antitoxins or analogues can be found throughout the studied 95 *M. catarrhalis* genomes. The numbers above each branch in the distance tree represent the bootstrap percentage, and the scale in the lower left corner corresponds to 0.1 nucleotide substitutions per site

A variety of potentially virulence-related genes were identified in the *M. catarrhalis* prophages (Table 2). The gene encoding virulence-associated protein E (VapE) was identified in Mcat19 and Mcat23. An antitoxin component highly related to *hicB*, the antitoxin component in the toxin-antitoxin pair *hicAB* [19], was identified in nine prophages. Further analysis showed that one major clade of antitoxin genes is found amongst the *M. catarrhalis* prophage antitoxins. Additionally, the prophage Mcat5 harbours up to three putative antitoxin genes (Fig. 5). The antitoxin components identified in our prophages were queried against all the *M. catarrhalis* genomes to look for *hicB* homologues (Fig. 5). The *hicB* homologue similar to BAP37890.1 was found 81 times in the 95 genomes, of which six were in complete prophage regions. These genes were found almost exclusively in the clade 1 prophages. The exception is the *hicB* homologue of the antitoxin in phage Mcat24 (clade 4 prophage), which was identified 83 times. Additionally, a truncated homologue of *hicB*, which is found in the prophages Mcat10, Mcat11, and Mcat12 (clade 2 prophages), was identified 24 times. An ORF corresponding to tellurium resistance and another ORF corresponding to a RecB exonuclease were identified.

**Table 2:** Putative virulence elements in *M. catarrhalis* prophages

Prophage	Putative virulence element	Query coverage	E-value	Identity	Accession number
Mcat1	Toxin-antitoxin system antitoxin component HicB [*Acinetobacter guillouiae*]	94 %	1e-21	62 %	BAP37890.1
Mcat2	Toxin-antitoxin system antitoxin component HicB [*Acinetobacter guillouiae*]	94 %	1e-21	62 %	BAP37890.1
Mcat3	ATP-dependent protease La [*Psychrobacter sp.* 1501(2011)]	97 %	0.0	72 %	WP_007395663.1
Mcat4	Toxin-antitoxin system antitoxin component HicB [*Acinetobacter guillouiae*]	85 %	4e-21	66 %	BAP37890.1
Mcat5	Toxin-antitoxin system antitoxin component HicB [*Acinetobacter guillouiae*]	94 %	1e-21	62 %	BAP37890.1
	Antitoxin HicB [*Yersinia pseudotuberculosis*]	100 %	2e-09	49 %	AIN15103.1
	Toxin-antitoxin system antitoxin component HicB [*Acinetobacter guillouiae*]	100 %	2e-20	65 %	BAP37890.1
Mcat6	Toxin-antitoxin system antitoxin component HicB [*Acinetobacter guillouiae*]	94 %	1e-21	62 %	BAP37890.1
Mcat10	Toxin-antitoxin system antitoxin component HicB [*Acinetobacter guillouiae*]	98 %	1e-18	56 %	BAP37890.1
Mcat11	Toxin-antitoxin system antitoxin component HicB [*Acinetobacter guillouiae*]	98 %	1e-18	56 %	BAP37890.1
Mcat12	Toxin-antitoxin system antitoxin component HicB [*Acinetobacter guillouiae*]	98 %	1e-18	56 %	BAP37890.1
Mcat17	Virulence-associated E family protein [*Nostoc punctiforme*]	85 %	4e-05	41 %	YP_001869297.1
Mcat21	Peptidase S41 [*Psychrobacter sp. G*]	99 %	0.0	62 %	YP_008162250.1
Mcat23	Virulence-associated E family protein [*Nostoc punctiforme* PCC 73102]	85 %	4e-05	41 %	WP_012412295.1
	Putative RecB exonuclease [*Acinetobacter* phage IME_AB3]	62 %	2e-06	52 %	YP_009008520.1
Mcat24	Toxin-antitoxin system antitoxin component HicB [*Acinetobacter guillouiae*]	94 %	1e-21	62 %	BAP37890.1
Mcat26	Integral membrane protein TerC family protein [*Helicobacter pylori*] involved in Te resistance	100 %	2e-36	96 %	WP_001934959.1
Mcat27	Type III restriction endonuclease subunit R [*Moraxella catarrhalis*]	100 %	3e-41	100 %	WP_004463103.1

Putative virulence elements identified from 32 *M. catarrhalis* prophages. The listed virulence element and associated accession number is taken from the BLASTP result with highest BLAST score

Two different protease genes were identified in Mcat3 and Mcat21. These proteases contained non-specific domains of varied functions, which are also found in a variety of organisms. The Mcat3 protease contains an ATPase associated with diverse cellular activities (AAA) domain, and two Lon domains. The Mcat21 protease contains a domain of unknown function, a structural PDZ domain, and an S41 serine endopeptidase domain; all of which are located in a Prc multi-domain region.

### Pan-genome analysis of *M. catarrhalis* prophages

**Fig. 6:**
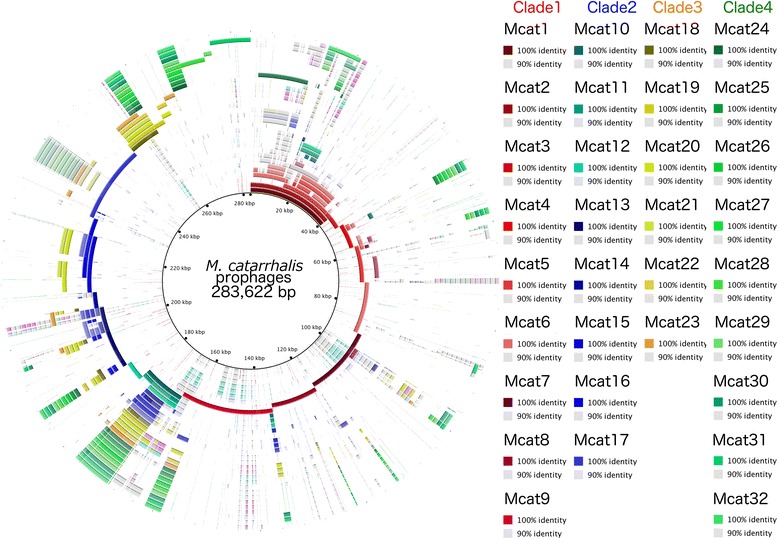
Pan-genome analysis of *M. catarrhalis* prophages. The pan-genome of 32 *M. catarrhalis* prophages is 283,622 bp in length, and is represented in the BRIG image above as the central black ring. Each concentric circle outwards from the reference pan-genome represents an individual prophage colour coded according to the assigned clades (red = clade 1, blue = clade 2, orange and yellow = clade 3, green = clade 4). A percentage identity cut-off above 90 % was used, which is represented by different hues in each ring (minimum of 90 % = grey, and up to maximum of 100 % = colour assigned to specific ring and prophage)

The pan-genome analysis of *M. catarrhalis* prophages is shown in Fig. 6. The innermost circle is the generated reference pan-genome against which each prophage genome is compared. Each concentric ring represents an individual prophage colour-coded according to the clade of origin as described above (clade 1 = red, clade 2 = blue, clade 3 = yellow / orange, and clade 4 = green, with each colour gradually fading as the BLAST identity score reaches 90 %). The reference pan-genome was found to be 283,622 bp in length, and describes a core-genome found in the majority of prophages, as well as an accessory genome more unique to individual prophages. The regions found in multiple rings (prophages) were compared to the PHAST annotation and were manually curated using BLAST against the NCBI database. They were found to code for various phage-related proteins, some virulence-related genes (as described above) or domains of unknown functions (data not shown).

### Distribution of prophages on a *M. catarrhalis* MLST backbone

**Fig. 7:**
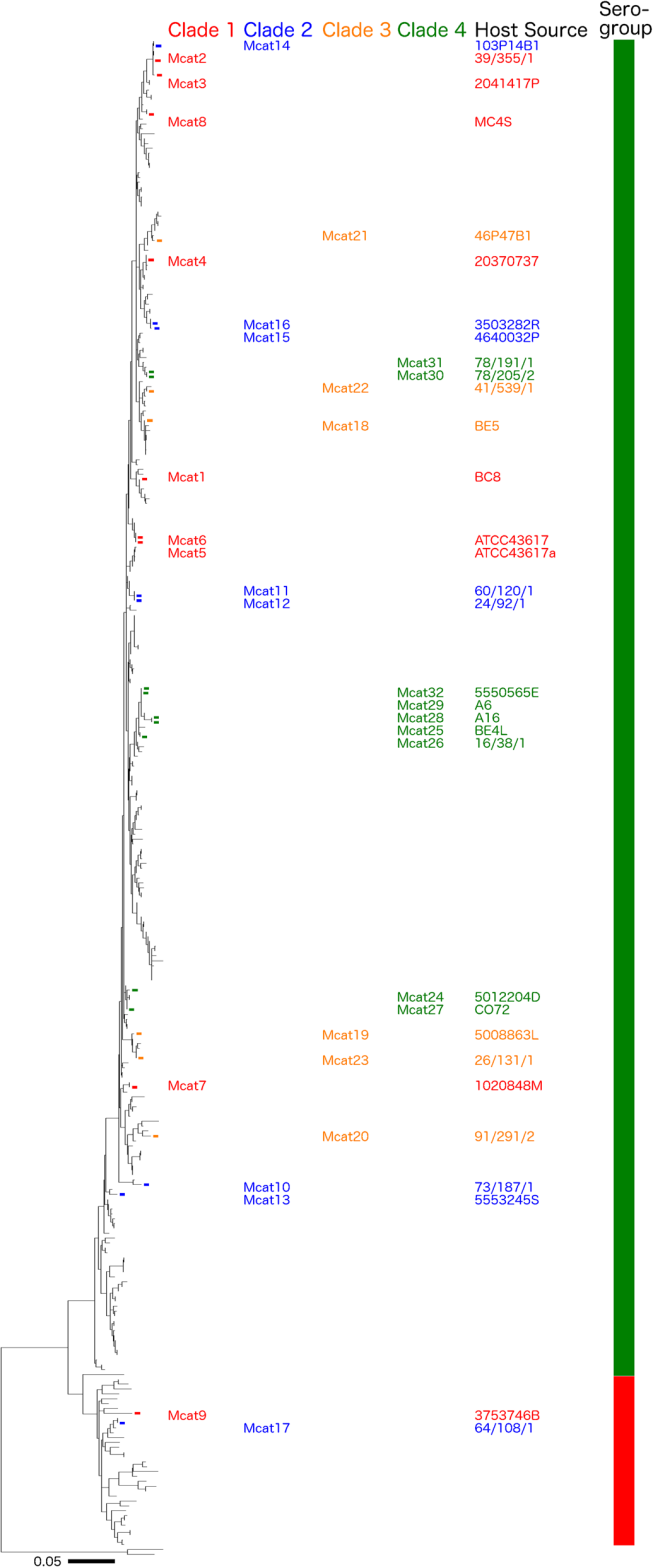
Distribution of prophages and prophage hosts on *M. catarrhalis* MLST phylogenetic tree. The phylogenetic tree based on *M. catarrhalis* MLST data is shown on the left. The two putative sero-groups based on MLST data are shown on the right where green = seroresistant and red = serosensitive. Each *M. catarrhalis* strain that hosts a complete prophage as described in this study is labelled according to colour of the prophage clade, red = prophage from clade 1, blue = clade 2, orange = clade 3, and green = clade 4. The scale on the lower left of image is equal to 0.05 nucleotide substitutions per site

*M. catarrhalis* strains have previously been grouped into two clades: a serosensitive lineage that is susceptible to serum complement and a seroresistant lineage that is not [9]. This study added strain data to the MLST backbone that shows the divergence of these two inferred phenotypic lineages, and plotted the presence of prophages onto the MLST backbone. The results indicated that the *M. catarrhalis* strains that host prophages are distributed randomly throughout this MLST backbone (Fig. 7).

## Discussion

Bacteriophages are the most abundant biological entities in the biosphere, infecting virtually every bacterial genus and species [20]. In environmental samples, phages are found in an order of magnitude more frequently than their hosts [21]. In light of their abundance and diversity, it is interesting that no *M. catarrhalis* prophages thus far have been identified. Preliminary work on the bacterium suggested the possibility of inducible prophages in *M. catarrhalis* strains (unpublished), however, attempts to induce and isolate *M. catarrhalis* phages were unsuccessful, leading to the choice of a bioinformatics approach to identifying *M. catarrhalis* prophages. Other studies have utilised bioinformatics approaches in the annotation of prophages in a diverse range of hosts, such as *Streptococcus suis* [16]*, Mycobacterium sp*. [22], and *Lawsonia intracellularis* [23]. Analysis of 95 *M. catarrhalis* assembled draft whole genome sequences resulted in the identification of 32 putative complete prophages, as well as numerous incomplete prophage remnants. These prophages, which have been assigned to the *Siphoviridae* family based on gene synteny and sequence identity, are annotated as ‘complete’ prophages according to the scoring system adopted by PHAST [24]. However, it is still unknown if they can excise from host genomes, form functional virions, and infect new host cells.

What qualifies as a prophage element has no definitive answer, as there is no comprehensive database for such elements, and no one gene is found in all prophages to serve as an identifying marker. However, it is expected that most bacterial genomes contain prophage elements, whether they are active prophages or remnants [15, 20, 25, 26]. Additionally, as prophage elements are continuously under degradative pressure [27], it was expected that there would be present more incomplete prophage elements, than questionable prophages, than complete prophages; even though the prophage remnants may still play roles in host function [28]. *M. catarrhalis*, with evolution spanning about 70 Ma, would be expected to have acquired, lost, modified, and inactivated a host of genetic parasites, including phages [9]. This order of expectation was found to be true over all the samples (131 incomplete prophages, 54 questionable prophages, and 32 complete prophages). Additionally, no lysogen harboured more than one prophage, as the presence of a complete prophage confers immunity to superinfection by other similar prophages [29, 30]. All the queried *M. catarrhalis* genomes had at least one incomplete prophage element except for the strains O35E, and 2041717D.

The discovered *M. catarrhalis* prophages fall into a narrow range of sizes ranging from 25 kb to 55 kb; *Siphoviridae* viruses have genome sizes usually ranging from 35 to 70 kb of DNA, with a mean of about 50 kb. The average GC content of the identified prophages was found to be 41.90 %, which is similar to that of the *M. catarrhalis* genome (~41 %). This is different from the average GC content of *Siphoviridae* (roughly 52.28 %, calculated from all 685 *Siphoviridae* sequences available in the NCBI database at the time of publication). However, the similarity of GC contents between identified prophages and host *M. catarrhalis* may be indicative of the prophages having incorporated into *M. catarrhalis* genomes early in the host’s speciation, or that the prophages have lain dormant in the host genomes.

An alignment of the putative attachment sites clusters them into a major and six minor groups (Additional file 6: Figure S4.). The clustering of the *att* sites did not correspond to the prophage clades, and did not directly reflect the diversity of integrase genes. However, it is known that various integrases can have secondary attachment sites that are divergent from the sequence of the primary attachment site, which may explain the variety of putative *att* sites [31–33]. Though the *att* sites are not identical, the conservation of a putative core site may relate to the hyperconservation of integrase genes [34–36].

Categorisation of prophages using global sequence and protein similarity has previously yielded reproducible and consistent results [37–39]. However, the reliability of such analyses has only been previously reported in the *Podoviridae* and *Myoviridae* families. This study found that a similar alignment and comparison approach classified 32 *M. catarrhalis* prophages as *Siphoviridae*, within a distinct group. Our prophage classification was reinforced by the analysis of head-neck-tail proteins using the programme Virfam [18]. The diversity of *M. catarrhalis* prophages is demonstrated in the breadth of categorisation using this software, where all prophages were categorised into four different Clusters of the Type I *Siphoviridae* (Additional file 2: Table S1).

The multiple alignment of all 32 *M. catarrhalis* putative prophages divides them into four separate clades. Based on the synteny and similarity of genes, prophage clades 2, 3, and 4 are highly related, but remain distinct, whereas clade 1 is more divergent than the others. This is illustrated by Virfam prophage categorisation, where 22 of 23 prophages in clades 2, 3, and 4 were categorised as Type 1, Cluster 3 *Siphoviridae* with high similarity to phages D3, HK97 and HK022 (the exception being Mcat28, which was categorised as a Type 1, Cluster 1 *Siphoviridae*). The clade 1 prophages were more variable, as described in the results section. Thus, clades 2, 3, and 4 represent a homogeneous and conserved group of *M. catarrhalis* prophages, but clade 1 represents a less well-defined cluster of *M. catarrhalis* prophages. Addition of more prophages to the distance tree would illustrate whether clade 1 requires further sub-division into more clades, or if a single clade is sufficient, particularly if the core genome for clade 1 prophages is smaller, or less homogeneous than that of clades 2, 3, and 4.

The BRIG alignment shown in Fig. 6 indicates that the prophages from clades 2, 3, and 4 are highly similar in their core genomes, and the core genome of clade 2 is a subset of that of clade 3, which in turn is a subset of the core genome of clade 4. The clade 1 prophages have a nearly unique core genome, represented by the span 0–40 kb of the reference pan-genome, which comprises the prophages Mcat1 – Mcat7. Mcat7, Mcat8 and Mcat9 are prophages with similarities to the clade 1 prophages, but also to the prophages of clades 2, 3, and 4, though they do not share the core genomes of the latter 3 clades.

The inclusion of five reference genera into the distance tree shows that the clades are adequately different to be considered for the categorisation of *M. catarrhalis* prophages; and that these prophages share some homology with the five genera of phages, but remain distinct as a group of their own. It is interesting to note that the highest region of identity between the reference genera and the *M. catarrhalis* prophages is centred around the tail fibre genes, which are involved in host recognition. We speculate that this may be a mechanism that confers host specificity to the phages.

This result is supported by the analysis of the more divergent genes for coat and tail shaft proteins. Coat genes clustered based on function into clusters representing 1) adaptor proteins, 2) joining and completion proteases, 3) connector proteins, 4) head proteases, and two undefined clusters, where there was little diversity in each cluster (Additional file 5: Figure S3). This distribution was observed for all *M. catarrhalis* prophages, regardless of prophage clade, indicating that selective pressure for the coat genes is different from that upon the integrase, terminase, portal, and plate genes. As with most structural genes, genetic mutation is more permissible than in non-structural genes, and as long as the function of the protein is preserved, there can be a larger amount of diversity before the gene renders its virus non-viable. Analysis of the tail shaft genes, however, presents a different picture. Although there are 7 clusters of genes, there are many more outlier genes or groups of genes that are significantly divergent from the major groups. Additionally, none of these groups could be assigned according to function, as there was no description of tail shaft genes in the reference genera. Furthermore, the distribution of genes from the reference genera puts these genes in closely associated clusters, with two of the 7 clusters of tail shaft genes consisting almost exclusively of genes from the reference genera. These distributions demonstrate diversity in *M. catarrhalis* prophage structural genes, which is expected for such genes under low selective pressure over a long evolutionary time period, such as that suggested by the analysis below.

The distribution of prophages and their *M. catarrhalis* hosts were compared based on the MLST tree created from 312 strains. There was no observable pattern correlating the prophage distribution and MLST backbone, which leads to the conclusion that the identified prophages are not confined to or fixed within a particular lineage of *M. catarrhalis* host. Furthermore, analysis of the prophage clades and how they are distributed amongst their hosts indicates that there is no observable pattern or relationship between prophage clade and *M. catarrhalis* host strain (Fig. 7). The data in this study suggest that the prophages have evolved alongside *M. catarrhalis* host before the divergence of serosensitive and seroresistant lineages, and that lateral transfer of the prophages between hosts is not limited by the divergence of the two *M. catarrhalis* lineages. The seroresistant lineage harbours prophages from all four prophage clades, but the serosensitive lineage only harbours prophages from clades 1 and 3. However, it is important to note that the serosensitive group is much smaller than the seroresistant group, and is only populated by 2 prophage hosts (as opposed to the 30 in the seroresistant group). This is compounded by the sequencing bias of this study in favour of the seroresistant lineage, where only four of the 95 isolates were found to be of the serosensitive lineage. The absence of any discernible pattern in this analysis demonstrates the diversity of *M. catarrhalis* prophages, supporting the prophage genetic analysis. However, further studies are required to establish any relationship between *M. catarrhalis* prophage and host, especially pertaining to *M. catarrhalis* isolates from the serosensitive lineage.

This diversity of *M. catarrhalis* prophages is contrasted by a hyperconservation of the phage specific genes encoding integrase, portal, and terminase (large and small subunits), which follows a general trend where the genes for clade 1 prophages cluster together, whereas those of clades 2, 3, and 4 cluster together (Fig. 3). This supports the argument that the prophages of clades 2, 3, and 4 share a relatively recent common ancestor, which is also reflected in the homogeneity of their core genome; whereas clade 1 prophages seem to have branched off earlier than clades 2, 3, and 4. Although the core genome of the clade 1 prophages shows a higher degree of diversity (Fig. 2 and Fig. 6), it is interesting that the integrase, portal, and terminase genes of the group 1 prophages are hyperconserved. This may support that the clade 1 prophages be categorised together, regardless of the near-absence of a core genome. The portal and two terminase genes can be grouped together as genes involved in packaging of new viral genetic material into a newly formed capsid. The generalised conservation of non-structural and portal genes in the two groups (clade 1 prophages compared to clade 2, 3, and 4 prophages) may indicate a difference in physiology of the two groups. This hypothetical evolutionary divergence is not reflected in the plate genes, which exhibit almost no diversity. This may be because the plate protein is essential for the injection of phage genetic material into the host, and significant mutation in the plate gene may render mutants incapable of infection and propagation. However, only 10 plate sequences were isolated, and it could be that this small sample size is indicative of plate genes that are too divergent from the database queries to be identified.

The integrase protein is spanned by two Pfam domains: DUF4102 and Phage_integrase, with the latter containing the active site. Further analysis of the ratio of non-synonymous to synonymous codons, d_N_/d_S_ values (also known as ω or K_a_/K_s_), and the trend of d_N_/d_S_ values averaged over 4 codons, for the integrase gene (Fig. 4) showed that the N-termini of the integrase genes (corresponding to DUF4102) are hyperconserved, with d_N_/d_S_ trends between 0.2 and 0.4, whereas the distal two thirds of the genes show conservation, where the d_N_/d_S_ trend only exceeds 1.0 at three points. Similar analysis of the large terminase subunit and portal genes revealed that the d_N_/d_S_ trend lines, averaged over 4 codons, for all genes are statistically significantly below 1.0, supporting a hypothesis of purifying selection for these genes. Such conservation suggests that the non-structural genes play a central and stable role in the maintenance of the phage.

The putative cargo genes identified in *M. catarrhalis* prophages are indirectly related to virulence. The majority of ORFs are related to antitoxin components of toxin-antitoxin systems, of which the most descriptive BLAST hit is *hicB*, the antitoxin component of the *hicAB* toxin-antitoxin system [19], and these antitoxins are most highly related to the *hicB* components in *Acinetobacter guillouaie* (BAP37890.1) and *Yersinia pseudotuberculosis* (AIN15103.1). Toxin-antitoxin systems have been shown to be involved in bacterial defence against phage infection via different mechanisms [40–42]. HicA expression is bacteriostatic, mediated by HicA cleavage of mRNA [43]. The HicAB toxin-antitoxin system is a type II system, where the HicB protein interacts sterically with HicA to inhibit its action. The corresponding toxin genes with sequence identity to *hicA* (BAP37889.1 and AIN15869.1) were not found using the BLAST and MUMmer [44] alignment algorithms. A Smith-Waterman alignment of the toxin and antitoxin components revealed that they share significant sequence similarity, but the relatively short toxin lengths may obscure results, leading to a reduced number of significant hits. Based on the presence of *hicB* antitoxin homologues in up to 87.4 % of the sequenced strains, it is suggested that at least one toxin-antitoxin system is utilised in *M. catarrhalis*, possibly relating to defence against phages. However, it could be that these are remnants of such systems, and have become degraded. Further research into the potential toxin-antitoxin systems related to *M. catarrhalis* prophages is under way.

Two prophages, Mcat23 and Mcat17 harboured genes similar to *vapE*. The virulence associated protein (Vap) family of virulence genes has been associated with clinically virulent strains of bacteria. The function of these virulence genes is not well described, but it is known that VapA is required in conjunction with other Vap proteins [45] in *Rhodococcus equi* for diversion of the phagosome-maturation pathway and prevents acidification of phagosomes [46]. With the exception of VapF, the Vap proteins are well conserved on the amino acid and nucleic acid levels, and share a well-conserved C-terminus [45, 47]. Although *vapE* is described to be present with *vapF* in virulence plasmids [48], *vap* genes have been shown to be widely distributed and also occur chromosomally as single genes [47].

## Conclusions

In conclusion, the approach used in this study has resulted in the first identification and characterisation of prophages in *M. catarrhalis.* The presence of prophages in host genomes does not prove functionality as active phages, but the distribution of similar prophages across different host lineages suggests that the prophages were, and may still be active. Induction of the prophages will be required to prove that active virions can be produced. This study demonstrates that along with significant diversity in *M. catarrhalis* prophages, there is unexpected hyperconservation of phage non-structural, as well as portal genes, which suggests that the preservation of these genes confers a physiological advantage and affects the fitness of *M. catarrhalis* host. The hyperconservation of phage-related genes may also indicate common ancestral (pro)phages for the *M. catarrhalis* prophages of clades 2, 3, and 4, as well as a common ancestral (pro)phage for the clade 1 prophages, which co-evolved with the bacterial species. Additionally, it is demonstrated that the prophages harbour virulence-associated genes, potentially playing a role in the physiology and virulence of *M. catarrhalis*.

## Methods

### Collection, sequencing and assembly of *M. catarrhalis* genomes

Eighty-five *M. catarrhalis* strains were collected for sequencing and analysis in this project, and an additional 12 sequences were obtained from the NCBI database (http://www.ncbi.nlm.nih.gov/) [15], detailed in Table 1. Forty-one strains were obtained from PathWest Laboratory Medicine WA, Australia; thirty-three strains were from the Kalgoorlie Otitis Media Research Project (KOMRP), Australia [49, 50]; 3 strains were from the Alexander Project [51]; 3 commensal strains were from Taiwan; three strains were from elsewhere in Australia; 1 strain was from Belgium; and 1 strain was from Holland [52]. This study included bacterial isolates for which no corresponding patient data were used. There is no requirement under Australian law to seek consent for the use of anonymised bacterial isolates for research. The strains were grown overnight on Brain Heart Infusion Agar (Oxoid) or Blood Agar (PathWest Media, P018) at 37 °C, and stored for up to 2 weeks at 4 °C. Whole plates of bacterial lawns were suspended in 180 μl ATL Buffer, and subsequently treated according to the Qiagen DNeasy blood & tissue protocol for purification of total DNA from animal tissues (spin-column protocol; Qiagen, Venlo). All strains were sequenced according to the Illumina Nextera-XT protocol, Revision C (Illumina, San Diego) [53]. This protocol was modified, where instead of using the Normalization Beads for the library normalisation step, the libraries were manually normalised using a 1:3 diluted library on an Agilent Technologies 2100 Bioanalyzer (Agilent Technologies, California) using a High Sensitivity DNA chip according to the Illumina Nextera (Illumina, San Diego) protocol [54]. This was performed to achieve a more uniform library index. Deep sequencing of the genomes was performed on an Illumina MiSeq sequencer (Illumina, San Diego) using 250 bp pair-end read chemistry and assembled *de novo* using the software Spades [55], where k-mer selection was optimised automatically.

### Identification of *M. catarrhalis* prophages

Prophages were identified in *M. catarrhalis* assembled genome sequences using the automated annotation programme PHAST [24]. PHAST assigns a score to putative prophages based on the presence of ‘cornerstone’ genes, phage-related genes, as well as the length of these genes. Putative prophages scoring between 90 and 150 are denoted as complete prophages, whereas those scoring between 60 and 90 are denoted questionable prophages, and those scoring below 60 are denoted incomplete prophages. Each ORF coding for phage-related genes was manually curated using BLASTP [56]. Complete prophage sequences were extracted from their respective *M. catarrhalis* genomes. ORFs 100 bp or larger were identified using Artemis, and selected for those opening with typical and atypical start codons [57]. These ORFs were uploaded as protein sequences to Virfam for prophage classification by analysis of phage-related proteins, and in particular head-neck-tail module genes [18].

### *M. catarrhalis* prophage similarity

All complete prophage genomes were diverse, such that they could not be aligned by simple nucleotide comparison. Instead, a less stringent block alignment was created using progressiveMauve [58]. A guide tree was visualised from the Mauve distance matrix using the programme FigTree 1.4.0. Regions of sequence identity between *M. catarrhalis* prophages, as well as archetypes from other Caudovirales phages were identified using the BLAST algorithm [59] from EasyFig 2.1 [60]. Additionally, each of the prophages that formed a clade were aligned and visualised using the BLAST algorithm from EasyFig 2.1 to show regions of sequence identity.

### Analysis of phage-related genes

Phage-related genes with similar functional annotation identified by PHAST were extracted from their respective prophage genomes, and aligned using ClustalW via MEGA 6.06 [61] and Unipro UGENE [62]. The *M. catarrhalis* prophage genes were compared to homologous genes in the five reference genera, λ, L5, N15, ϕC31 and Tuna viruses, as well as homologous genes from closely related species: c341, ϕ297, ϕ80, and HK022. The nucleotide alignments of conserved genes were used for construction of distance trees using the Maximum Likelihood method using MEGA 6.06 with the following parameters: bootstrap value of 1000, Tamura-Nei model, a uniform rate of substitution amongst sites, and inferred using Nearest-Neighbor-Interchange heuristic method applied to an initial Neighbor-Joining/BioNJ tree. These trees were confirmed using the Bayesian analysis of the MrBayes [63] utility available in Unipro UGENE, in which a substitution model of HKY85 was used (Nst = 2) with default settings and a random seed number. For genes too diverse to be aligned and compared at the nucleotide level, translated amino acid alignments were also used, and this was constructed using the ClustalW feature of Unipro UGENE. For genes too divergent to be aligned via Maximum Likelihood method (coat and tail genes), translated protein alignments were used to construct Neighbor-Joining trees in Unipro UGENE using the following parameters; a distance matrix model of F84, transition/transversion ratio of 2.0, and a bootstrap (replicate) value of 1000. These trees were confirmed using the Bayesian analysis of the MrBayes utility available in Unipro UGENE, in which a substitution model of HKY85 was used (Nst = 2) with default settings and a random seed number [63]. The per codon d_N_/d_S_ ratios (ω) for the integrase, large terminase and portal genes were computed as a by-product of creating Bayesian phylogenetic trees from the respective genes using MrBayes [63], where d_N_/d_S_ was allowed to vary across codons. A gamma model was used for variation between sites, together with a proportion of invariant sites. Mean d_N_/d_S_ values were reported for each triplet and then input to Excel to create the graph, including trend-line.

### Pan-genome analysis of *M. catarrhalis* prophages

The sequences for all *M. catarrhalis* prophages were used for a pan-genome comparison. To create a reference pan-genome of the prophages, the programme Panseq was run utilising default parameters [64]. Each prophage genome was aligned against this reference genome using the BLAST [56] algorithm found with BLAST Ring Image Generator (BRIG) [65] with the following parameters: all *M. catarrhalis* prophages represented a unique ring, except for the annotations for Mcat5 and Mcat6, from strains ATCC43617 and ATCC43617a, which were combined into a single output to avoid redundancy; an identity threshold between 90 and 100 % was selected for the image colour gradient generation; a default minimum threshold of 50 % was used for alignment; phages were ordered and colour coded according to their respective clades as described in the results section; and the output image was scaled to include a legend.

### Extraction of multi-locus sequence type backbone

MLST data for eight housekeeping genes, *abcZ*, *adk*, *efp*, *fumC*, *glyβ*, *mutY*, *ppa*, and *trpE,* were extracted from the assembled genomes. The gene sequences were concatenated and the concatenation was used to construct a phylogenetic tree similar to that performed by Wirth et al. in 2007 [9]. The sequences were aligned and a tree was created using MEGA6.06 using the following parameters: a Nearest-Neighbour Interchange model was imposed on an initial Neighbour Joining (NJ/BioNJ) tree to create a Maximum-Likelihood tree modelled on protein-coding nucleotide sequences, with a bootstrap value of 1000, using a Tamura-Nei model, and a uniform rate of substitution amongst sites [61]. The dataset of MLST genes and strains includes 312 strains of *M. catarrhalis*, of which 95 strains are from this study, 12 from Davie et al. [15], and the remainder from Wirth et al. [9].

## Additional files

Additional file 1: Figure S1. PHAST annotation of 32 *M. catarrhalis* prophages. 32 *M. catarrhalis* prophages were annotated as complete prophages by the PHAST programme, defined as such for scoring between 90 and 150 on the PHAST scoring system. Each arrow represents an open reading frame found in each prophage, colour coded as in the legend below each column: tail fibre (dark green), terminase (light blue), coat (dark blue), integrase (red), plate (purple), portal (dark orange), tail shaft (light green), hypothetical protein (navy blue), and other phage-like protein (light orange). Each prophage is named according to the methods section, and this name is colour coded according to the prophage clade of origin as per Fig. 1: clade 1 (red), clade 2 (dark blue), clade 3 (orange), and clade 4 (dark green). (TIFF 6959 kb) Additional file 2: Table S1. PHAST and Virfam results for 32 *M. catarrhalis* prophages. ^1^ 32 *M. catarrhalis* prophages were annotated as complete prophages by the PHAST programme, defined as such for scoring between 90 and 150 on the PHAST scoring system. ^2^ Virfam analysis for categorisation of the prophages designated a family, type, and cluster for each prophage, along with a phylogenetic tree depicting the relationship of the *M. catarrhalis* prophage head-neck-tail modules with those of known phages in the Aclame database. (DOCX 143 kb) Additional file 3: Figure S2. Alignment of *M. catarrhalis* prophages of clade 2 (Mcat10–Mcat17, dark blue), clade 3 (Mcat18–Mcat23, orange), and clade 4 (Mcat24–Mcat32, dark green). Each horizontal line represents a prophage, and each arrow represents an open reading frame (ORF) colour coded according to function: terminase (light blue), portal (dark orange), protease (yellow), coat (dark blue), tail shaft (light green), integrase (red), other phage-related protein (light orange), tail fibre (dark green), and plate (purple). Vertical lines between prophages shows aligned regions whose hues of grey correspond to the percentage identity of aligned regions as specified in the spectrum at the bottom right of each image. Image generated by EasyFig [60]. (TIFF 4381 kb) Additional file 4: Table S2. Presence of phage-related genes in analysed *M. catarrhalis* prophages. The detection of phage-related genes in analysed *M. catarrhalis* prophages using the programme PHAST is signified with presence (+) or absence (−). ^1^The assignment of prophages to clades 1 to 4 is as described in methods section. (DOCX 122 kb) Additional file 5: Figure S3. Distance tree of translated coat nucleotide sequences of *M. catarrhalis* prophages. Six groups are identified, four of which are clustered according to function: (red) head adaptor protein, (dark blue) joining and completion protease, (dark green) connector protein, and (purple) head protease. Alignment was constructed using ClustalW algorithm, and tree generation with Neighbor-Joining method using Unipro UGENE programme. The schematic of a *Siphoviridae* phage is presented on the right with labels for phage-related structures and 100nm scale [66]. (TIFF 141 kb) Additional file 6: Figure S4. Clustering of *M. catarrhalis* prophage attachment sites. Twenty-three *att* sites are identified, which cluster into a major group and 6 minor groups. Each *att* site is labelled according to prophage of origin, which is coloured according to the relevant prophage clade in Fig. 1 (red = clade 1, dark blue = clade 2, orange = clade 3, and green = clade 4). The nucleotide alignment is shown on the right (green A = adenine, red T = thymine, blue C = cytosine, purple G = guanine), and scale at bottom left indicates 10 nucleotide substitutions per site. (TIFF 3747 kb)
