# Supplementary material for: Novel Moraxella catarrhalis prophages display hyperconserved non-structural genes despite their genomic diversity
Source: BMC Genomics. 2015 Oct 24;16:860. doi: 10.1186/s12864-015-2104-1 (PMC4619438; doi:10.1186/s12864-015-2104-1)
Supplement: Additional file 2: Table S1. — PHAST and Virfam results for 32 M. catarrhalis prophages. 1 32 M. catarrhalis prophages were annotated as complete prophages by the PHAST programme, defined as such for scoring between 90 and 150 on the PHAST scoring system. 2 Virfam analysis for categorisation of the prophages designated a family, type, and cluster for each prophage, along with a phylogenetic tree depicting the relationship of the M. catarrhalis prophage head-neck-tail modules with those of known phages in the Aclame database. (DOCX 143 kb) [file 12864_2015_2104_MOESM2_ESM.docx]

| Prophage | PHAST score^1^ | Family^2^ | Type^2^ | Cluster^2^ | Phage similarity^2^ |
| --- | --- | --- | --- | --- | --- |
| Mcat1 | 150 | *Siphoviridae* | 1 | 3 | φ4795 |
| Mcat2 | 150 | *Siphoviridae* | 1 | 3 | φ4795 |
| Mcat3 | 120 | *Siphoviridae* | 1 | 3 | φ4795 |
| Mcat4 | 150 | *Siphoviridae* | 1 | 3 | φ4795 |
| Mcat5 | 150 | *Siphoviridae* | 1 | 4 | PBl1 |
| Mcat6 | 150 | *Siphoviridae* | 1 | 1 | SPP1 group |
| Mcat7 | 150 | *Siphoviridae* | 1 | 3 | φ4795 |
| Mcat8 | 150 | *Siphoviridae* | 1 | 5 | φC31, φBT1, φHSIC, XP15, K57, SETP3, M6 |
| Mcat9 | 150 | *Siphoviridae* | 1 | 3 | φ4795 |
| Mcat10 | 150 | *Siphoviridae* | 1 | 3 | D3, HK97, HK022 |
| Mcat11 | 150 | *Siphoviridae* | 1 | 3 | D3, HK97, HK022 |
| Mcat12 | 150 | *Siphoviridae* | 1 | 3 | D3, HK97, HK022 |
| Mcat13 | 150 | *Siphoviridae* | 1 | 3 | D3, HK97, HK022 |
| Mcat14 | 100 | *Siphoviridae* | 1 | 3 | D3, HK97, HK022 |
| Mcat15 | 150 | *Siphoviridae* | 1 | 3 | D3, HK97, HK022 |
| Mcat16 | 100 | *Siphoviridae* | 1 | 3 | D3, HK97, HK022 |
| Mcat17 | 150 | *Siphoviridae* | 1 | 3 | D3, HK97, HK022 |
| Mcat18 | 110 | *Siphoviridae* | 1 | 3 | D3, HK97, HK022 |
| Mcat19 | 100 | *Siphoviridae* | 1 | 3 | D3, HK97, HK022 |
| Mcat20 | 120 | *Siphoviridae* | 1 | 3 | D3, HK97, HK022 |
| Mcat21 | 120 | *Siphoviridae* | 1 | 3 | D3, HK97, HK022 |
| Mcat22 | 130 | *Siphoviridae* | 1 | 3 | D3, HK97, HK022 |
| Mcat23 | 130 | *Siphoviridae* | 1 | 3 | D3, HK97, HK022 |
| Mcat24 | 150 | *Siphoviridae* | 1 | 3 | D3, HK97, HK022 |
| Mcat25 | 130 | *Siphoviridae* | 1 | 3 | D3, HK97, HK022 |
| Mcat26 | 130 | *Siphoviridae* | 1 | 3 | D3, HK97, HK022 |
| Mcat27 | 110 | *Siphoviridae* | 1 | 3 | D3, HK97, HK022 |
| Mcat28 | 150 | *Siphoviridae* | 1 | 1 | SPP1 group |
| Mcat29 | 130 | *Siphoviridae* | 1 | 3 | D3, HK97, HK022 |
| Mcat30 | 100 | *Siphoviridae* | 1 | 3 | D3, HK97, HK022 |
| Mcat31 | 100 | *Siphoviridae* | 1 | 3 | D3, HK97, HK022 |
| Mcat32 | 150 | *Siphoviridae* | 1 | 3 | D3, HK97, HK022 |

**Supplementary 1: PHAST and Virfam results for 32 *M. catarrhalis* prophages.**

^1^ 32 *M. catarrhalis* prophages were annotated as complete prophages by the PHAST programme, defined as such for scoring between 90 – 150 on the PHAST scoring system. ^2^ Virfam analyisis for categorisation of the prophages designated a family, type, and cluster for each prophage, along with a phylogenetic tree depicting the relationship of the *M. catarrhalis* prophage head-neck-tail modules with those of known phages the Aclame database.
