# Supplementary material for: Novel Moraxella catarrhalis prophages display hyperconserved non-structural genes despite their genomic diversity
Source: BMC Genomics. 2015 Oct 24;16:860. doi: 10.1186/s12864-015-2104-1 (PMC4619438; doi:10.1186/s12864-015-2104-1)
Supplement: Additional file 4: Table S2. — Presence of phage-related genes in analysed M. catarrhalis prophages. The detection of phage-related genes in analysed M. catarrhalis prophages using the programme PHAST is signified with presence (+) or absence (−). 1The assignment of prophages to clades 1 to 4 is as described in methods section. (DOCX 122 kb) [file 12864_2015_2104_MOESM4_ESM.docx]

| Clade^1^ | Phage name | Integrase | Plate | Terminase  large subunit | Terminase  small subunit | Portal | Tail fiber | Tail shaft | Coat |
| --- | --- | --- | --- | --- | --- | --- | --- | --- | --- |
| 1 | Mcat1 | + | + | + | + | + | + | + | + |
|  | Mcat2 | + | + | + | + | + | + | + | + |
|  | Mcat3 | + | - | + | + | + | - | + | + |
|  | Mcat4 | + | + | + | + | + | + | + | + |
|  | Mcat5 | - | + | + | + | + | + | + | + |
|  | Mcat6 | + | - | + | + | + | + | + | + |
|  | Mcat7 | - | + | + | - | + | + | + | + |
|  | Mcat8 | + | + | + | - | + | + | + | + |
|  | Mcat9 | + | - | + | - | + | + | + | + |
| 2 | Mcat10 | + | - | + | + | + | + | + | + |
|  | Mcat11 | + | - | + | + | + | + | + | + |
|  | Mcat12 | + | - | + | + | + | + | + | + |
|  | Mcat13 | + | - | + | + | + | + | + | + |
|  | Mcat14 | + | - | + | + | + | - | + | + |
|  | Mcat15 | + | + | + | + | + | + | + | + |
|  | Mcat16 | + | + | + | + | + | + | + | + |
|  | Mcat17 | + | + | + | + | + | + | + | + |
| 3 | Mcat18 | + | - | + | + | + | + | + | + |
|  | Mcat19 | + | - | + | + | + | + | + | + |
|  | Mcat20 | - | - | + | + | + | - | + | + |
|  | Mcat21 | - | - | + | + | + |  | + | + |
|  | Mcat22 | + | - | + | + | + | + | + | + |
|  | Mcat23 | - | - | + | + | + | - | + | + |
| 4 | Mcat24 | - | + | + | + | + | + | + | + |
|  | Mcat25 | - | - | + | + | + | + | + | + |
|  | Mcat26 | - | - | + | + | + | + | + | + |
|  | Mcat27 | + | - | + | + | + | + | + | + |
|  | Mcat28 | + | - | + | + | + | + | + | + |
|  | Mcat29 | - | - | + | + | + | + | + | + |
|  | Mcat30 | - | - | + | + | + | - | + | + |
|  | Mcat31 | - | - | + | + | + | - | + | + |
|  | Mcat32 | - | - | + | + | + | - | + | + |

**Supplementary 3: Presence of phage-related genes in analysed *M. catarrhalis* prophages.**

The detection of phage-related genes in analysed *M. catarrhalis* prophages using the programme PHAST is signified with presence (+) or absence (-). ^1^The assignement of prophages to clades 1 to 4 is as described in methods section.
