# Supplementary figures and images for: Novel Moraxella catarrhalis prophages display hyperconserved non-structural genes despite their genomic diversity
Source: BMC Genomics. 2015 Oct 24;16:860. doi: 10.1186/s12864-015-2104-1 (PMC4619438; doi:10.1186/s12864-015-2104-1)

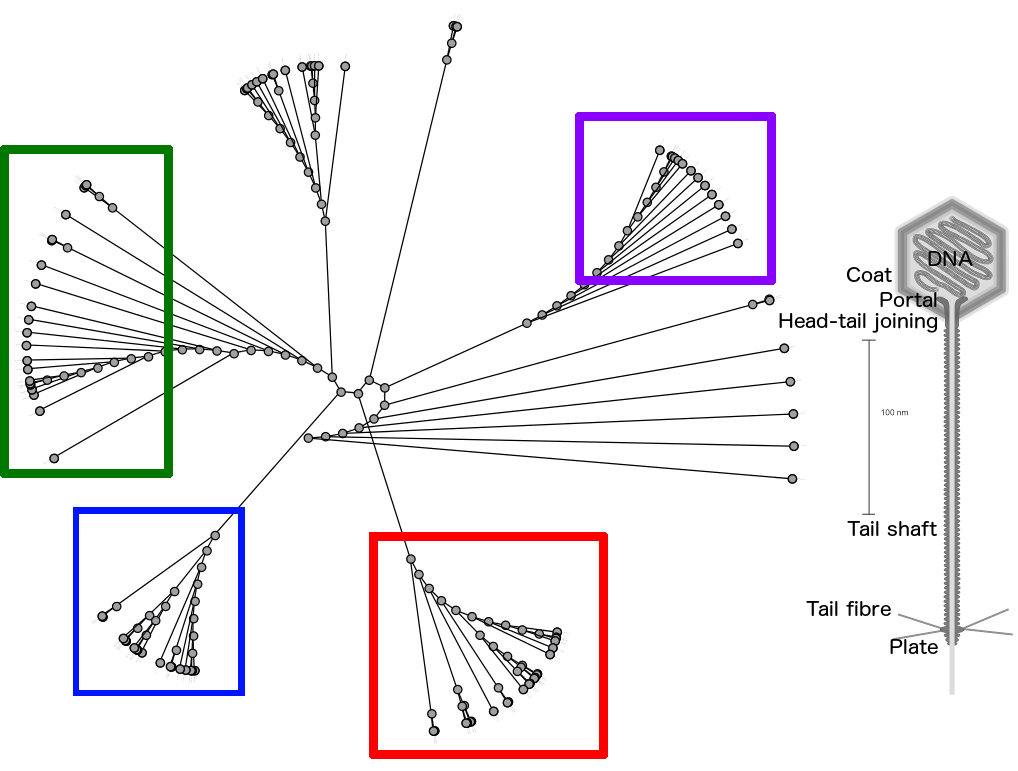

Supplement: Additional file 5: Figure S3. — Distance tree of translated coat nucleotide sequences of M. catarrhalis prophages. Six groups are identified, four of which are clustered according to function: (red) head adaptor protein, (dark blue) joining and completion protease, (dark green) connector protein, and (purple) head protease. Alignment was constructed using ClustalW algorithm, and tree generation with Neighbor-Joining method using Unipro UGENE programme. The schematic of a Siphoviridae phage is presented on the right with labels for phage-related structures and 100nm scale [66]. (TIFF 141 kb) [file 12864_2015_2104_MOESM5_ESM.tif]

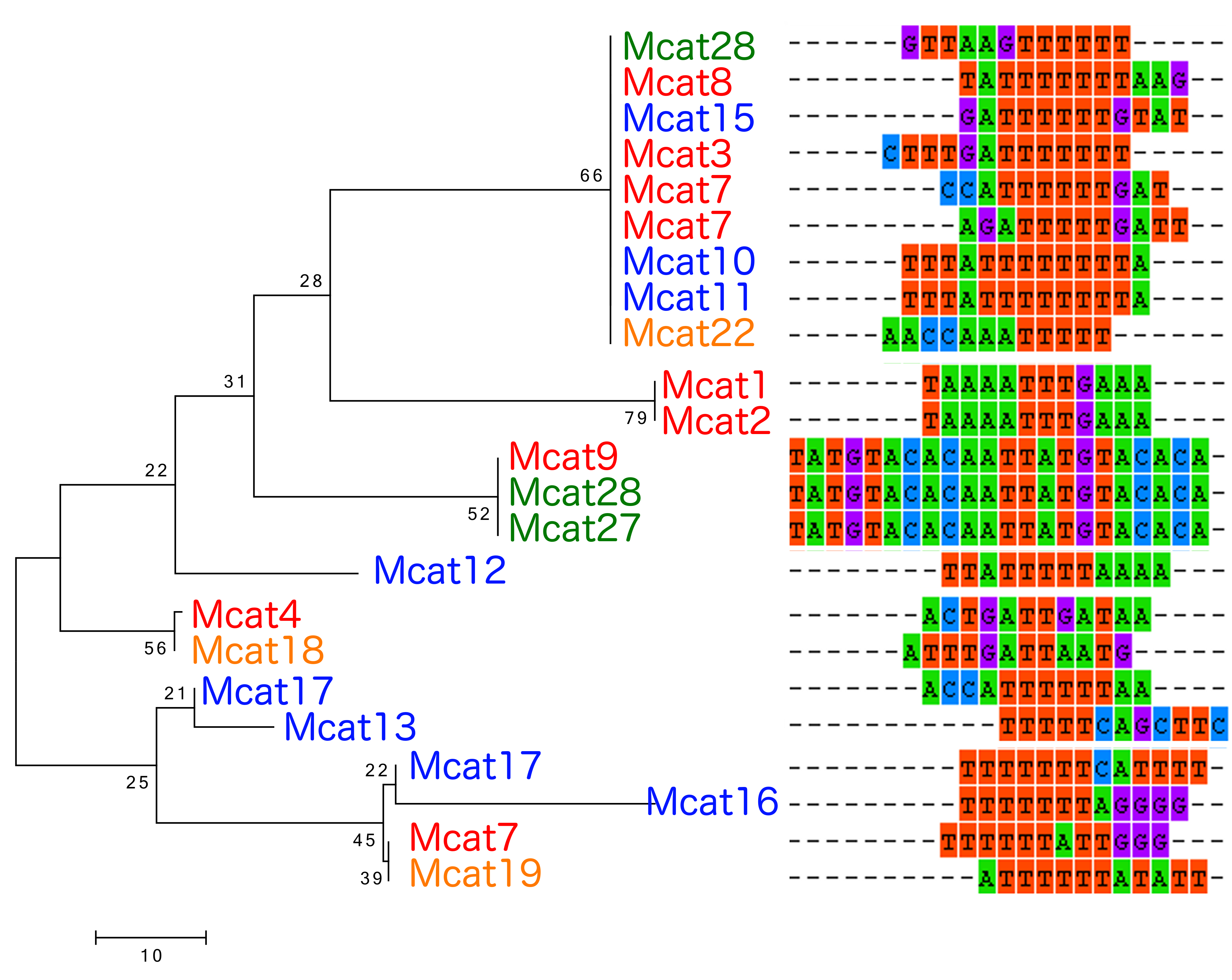

Supplement: Additional file 6: Figure S4. — Clustering of M. catarrhalis prophage attachment sites. Twenty-three att sites are identified, which cluster into a major group and 6 minor groups. Each att site is labelled according to prophage of origin, which is coloured according to the relevant prophage clade in Fig. 1 (red = clade 1, dark blue = clade 2, orange = clade 3, and green = clade 4). The nucleotide alignment is shown on the right (green A = adenine, red T = thymine, blue C = cytosine, purple G = guanine), and scale at bottom left indicates 10 nucleotide substitutions per site. (TIFF 3747 kb) [file 12864_2015_2104_MOESM6_ESM.tif]
